# Trusting Wisely? Developmental Changes in How Children Learn and Adapt to Partner Trustworthiness

**DOI:** 10.1111/desc.70205

**Published:** 2026-04-26

**Authors:** Yiyan Rose Wang, Felix Warneken

**Affiliations:** ^1^ Department of Psychology University of Michigan Ann Arbor Michigan USA

**Keywords:** reinforcement learning, social cognition, trust game, trustworthiness

## Abstract

Trusting selectively is a crucial ability in successfully navigating social environments: trusting a trustworthy partner maximizes mutual benefits; withholding trust from an untrustworthy partner minimizes chances of being exploited. Here, we ask how children's trust expectations and experienced trustworthiness inform their own trust decisions. Using a child‐friendly repeated Trust Game, we employ a computational modeling approach to examine how children learn about the trustworthiness of others through experience and adjust their own trust behaviors. We tested *N* = 96 children ages 6–11 years who played 40 trials of the game as trustors with a trustworthy and an untrustworthy trustee. Regression analyses showed that overall, children shared more with trustworthy than untrustworthy partners and older children shared overall more than younger children. Computational models provided a more fine‐grained analysis of the learning pattern: a reinforcement learning model that included parameters for individual variation in prior trust expectations and a parameter quantifying the behavioral adjustment over time provided the best fit for the data. Using this computational approach, we could ascertain that children's behavioral adjustments became less responsive over time, suggesting a shift toward more stable patterns of trust once initial impressions were formed. Together, these findings shed light on the development of children's trust interaction with novel partners and highlight the opportunity that computational modeling provides in capturing developmental differences in trust behaviors.

**Summary:**

- Children aged 6–11 used experience to distinguish trustworthy from untrustworthy partners in a repeated Trust Game, sharing more with the trustworthy than the untrustworthy partner.
- Older children shared more than younger children, although they were not significantly differentiating between the trustworthy than the untrustworthy partner.
- Reinforcement learning modeling suggested that children updated trust expectations from experience, with age‐related differences in the efficiency of impression updating.
- These findings highlight both children's capacity in flexibly adjusting their economic trust behaviors and the value of computational modeling in studying economic trust development.

## 1 Introduction

Trust is crucial in enabling and maintaining interpersonal relationships. From children relying on caregivers to keep them safe to adults depositing their savings in banks, people willingly put themselves in vulnerable positions with the expectations that others will act in reliable and cooperative ways. Yet, trusting can be risky: misplacing trust on someone untrustworthy leads to the possibility of being exploited. Therefore, the ability to detect a partner's trustworthiness and adjust one's own trust decisions accordingly is a critical adaptive social skill for individuals to successfully navigate their social environments. How does this ability develop across development? Although research in the past few decades has extensively studied economic trust behaviors in adults, little research has focused on the psychological mechanisms behind this phenomenon in young children, with only emerging research suggesting crucial changes in economic trust behaviors in early childhood. The current study addresses this gap by using experimental and computational approaches to understand how young children learn about the trustworthiness of others through experience. Specifically, we examined whether and how young children adjust their own behaviors when they interact with novel, trustworthy or untrustworthy partners.

On the most general level, trust requires individuals to demonstrate a “willingness to accept vulnerability” (Rousseau et al. 1998). Here, we focus on economic trust as a core feature of cooperative exchanges that involve sharing of resources. Research with adults shows that individuals trust by voluntarily investing resources in social interaction partners, with the expectation that they will reciprocate at a later time (Fehr 2009). However, since there is no explicit guarantee that others would reciprocate, trusting is a risky choice that makes the trustor vulnerable. While inherently risky, economic trust is not simply a decision‐making process under risk because it occurs as a social exchange with other individuals, including the risk of being betrayed by others (rather than just losing a bet or dice roll; Fetchenhauer and Dunning 2012). Past research with adults most often used an experimental task called Trust Game or Investment Game, as a behavioral measure of economic trust. In this game, trustors decide whether they want to invest their own resources to trustees, who in turn have the opportunity to reciprocate or abuse the trust they received (Berg et al. 1995). A wide range of studies have used this method and found that across cultures, adults trust others in the trust game at least to some degree, although the absolute amount varies (e.g., Balliet and Van Lange 2013; Johnson and Mislin 2011). Moreover, trustees reciprocate that trust by sharing some of their profit with the trustor. The prevalence of economic trust across the globe underscores the important function it serves in social relationships and the need to understand its development.

### 1.1 Economic Trust in Children

Despite the abundance of research on economic trust in adults, little research has investigated the origins of economic trust in development. Economic trust allows children to establish and maintain long‐term cooperative relationships that would be beneficial for development, especially if that trust is placed on those who are trustworthy. Do young children show selective economic trust? Theory and evidence suggest that while economic trust may emerge early in development, selective trust may emerge later as children gain more complex cognitive skills to recognize cues for reciprocity. Warneken (2018) argued that the two challenges in cooperation that children need to solve are how to generate and distribute benefits, with the first challenge rooted in evolutionary history and the second challenge developed later on in ontogeny. Here we argue that trust plays a central role in resolving both challenges: when initiating cooperation to generate resources, children must trust that others will reciprocate effort and not exploit their contributions; when distributing shared resources, trust enables children to anticipate fairness in outcomes and to accept temporary inequality with expectation of future reciprocity. In situations where chances for future reciprocity are uncertain, it is possible that generally trusting young children would not be able to trust selectively, lending themselves more vulnerable to betrayal.

Some initial findings support the intuition that in resource‐sharing contexts, the capacity to trust emerges earlier than the ability to trust selectively, potentially as children become more sensitive to social and contextual cues. For example, Margoni et al. (2022) found that when presented with information about partners’ past social and moral behaviors (helper versus hinderer, leader versus bully), 7–8‐year‐olds showed more economic trust toward morally positive partners than negative ones. By contrast, 4–5‐year‐olds did not show significant differences in how likely they trusted the prosocial versus antisocial partners, although they trusted leaders more than they trusted bullies, suggesting a developing sensitivity to sociomoral information in early school years. Moreover, older children are also increasingly attuned to the possibility of reciprocity: compared to 4–5‐year‐olds, 9–10‐year‐old children invested less when their partners were tempted to betray them, and more when they knew that their trustee was trustworthy (Evans et al. 2013).

One confound in this set of findings, though, is that from the experimental design employed in these studies, economic trust as an investment of resources with others in anticipation of a mutualistic benefit could not be distinguished from generosity as unilateral sharing of valuable resources with others. To address this concern, Rosati et al. (2019) designed a developmentally appropriate version of the Trust Game and showed that both 4‐ and 6‐year‐olds understood the difference between trust and generosity as they passed on more resources to their partners when their trust could be reciprocated. Moreover, after learning about the two partners’ past trustworthiness, 6‐year‐olds were significantly more likely to trust the trustworthy partner than the untrustworthy one, while 4‐year‐olds only showed a trend to differentiate between the partners. Lastly, not only do children recognize the possibility of reciprocity when deciding whether to trust their partner with resources, they also understand the need to reciprocate when being trusted. A recent study found that 5–8‐year‐old children reciprocated more resources when they knew that the resource came from trust as compared to generosity (Amir et al. 2021).

Overall, past findings suggest an early emergence and increasingly sophisticated nature of economic trust in childhood. Less is known, however, about the psychological mechanisms that enable children to trust selectively based on their own trust expectations and personal experience of interacting with trustworthy and untrustworthy partners over time.

### 1.2 Trust Over Repeated Interactions

Relationships unfold over time. The ability to trust in a single interaction is not sufficient to maintain cooperative relationships in the long run. Therefore, in order to sustain cooperation and avoid being exploited, people need to keep track of the trustworthiness of their partners and selectively trust those who are likely to reciprocate. Researchers often use repeated Trust Games to simulate this dynamically evolving nature of relationships to examine how trust behaviors change over time (e.g., Engle‐Warnick and Slonim 2006). Behavioral economics predicts that, assuming that all individuals are rational decision makers who want to maximize their own profit, trust is theoretically most likely to emerge in repeated games with no clearly defined endpoint, where future interactions are salient and both the trustor and the trustee have incentives to maintain cooperation. In contrast, trust is more fragile in one‐shot settings, where betrayal carries no future consequence (e.g., Bó 2005).

Our key question concerns whether and how children adapt their behavior based on their partner's actions in repeated interactions. Through experience, people can learn and update their impressions of their partner's trustworthiness and use those impressions to their benefit by increasingly trusting the ones who reciprocate and distrust the ones who betray. Research has just started to examine this adaptation process in childhood. Rosati et al. (2019) first introduced 4‐ and 6‐year‐old children to one trustworthy and one untrustworthy partner and then had them play eight trials of the Trust Game with each partner. Results suggested that 6‐year‐olds’ trust toward the trustworthy partner increased whereas their trust toward the untrustworthy partner decreased over eight trials. By contrast, 4‐year‐olds were not able to appropriately adjust their behaviors: in fact, their trust for both partners decreased over trials, potentially because they overgeneralized distrust to both partners after experiencing betrayal from one. This research points to early childhood as a critical time window when children show rapid development in their ability to not only place economic trust in others but also trust selectively.

More recent work highlights the role of expectations in how children's economic trust decisions unfold over repeated interactions. For example, Siddique et al. (2022) found that 8‐ to 10‐year‐old children's trust in a repeated Trust Game was initially based on the perceived facial trustworthiness of their partners: they invested more tokens in trustworthy‐looking partners compared to untrustworthy‐looking partners. As they experienced fair versus unfair returns of their investment in the repeated Trust Game, children overcame the initial facial trustworthiness bias and used experienced trustworthiness to guide their trust decisions, trusting the fair partners more than the unfair partners. Together, past research suggests that children draw on both prior beliefs and actual experience to guide their economic trust decisions, although the manner in which experience gets integrated is opaque and the age in which this adaptation process reliably occurs is unclear. Importantly, these prior paradigms have used partners with fixed, consistent behavior. Whether children can calibrate their trust selectively when partner behavior is variable remains an open question, with important implications for ecological validity given that real‐world social partners can be inconsistent.

### 1.3 Computational Modeling of Trust

Computational modeling offers important insights into the underlying psychological processes in observed behavioral data. Amongst different models, reinforcement learning models are particularly suitable for understanding how social decision‐making, like economic trust, unfolds through repeated interaction between the decision‐maker and their environment (Sutton and Barto 1998). The core idea behind reinforcement learning is that learning is driven by the differences between expectation and actual outcomes, assuming that the decision‐maker is motivated to maximize their reward on each trial. In the context of trust decisions, a learning rate (*λ*) reflects the extent to which expectations about a partner's trustworthiness is updated as a proportion of the difference between expected and experienced trustworthiness. Experiencing a reward greater than expected would lead individuals to increase their expectations about a partner's trustworthiness and vice versa. Moreover, reinforcement learning models allow for modeling of decision priors (*p*), which can be interpreted as the baseline expectations of trustworthiness that would bias individuals’ decisions toward trusting or distrust. By estimating group‐level reinforcement learning parameters, we can gain a more nuanced understanding of how trust decisions unfold over time that cannot be captured under traditional regression analyses.

Research that used reinforcement learning to model economic trust behaviors in adults and older children has highlighted how prior trust expectations and experience interact to inform trust behavior adjustment over time. For example, Chang et al. (2010) showed that both initial perceived facial trustworthiness and experienced trustworthiness influenced adults’ trust decisions in a repeated Trust Game. The reinforcement learning model that best fit participants’ behaviors captured a dynamic belief‐updating process: after each interaction, individuals revised their expectations about a partner's trustworthiness and used those updated beliefs to guide future decisions. Crucially, this model outperformed alternatives like a gain‐loss model that only considered asymmetric learning between gains and losses or a confirmation bias model that assumed initial trustworthiness expectations dictate how subsequent experience is interpreted. By allowing researchers to directly compare these alternatives using the same data, reinforcement learning methods provide a mechanistic explanation that trust unfolds not as a series of reflexive reactions or as a fixed bias, but as a dynamic belief updating process.

Although studies with adults show what kind of reinforcement learning applies for a given task, the learning process itself can undergo meaningful changes across development. Computational models are particularly helpful in shedding light on how learning changes with age. For example, Nussenbaum et al. (2023) showed that when exploring under uncertainty, adolescents and adults showed attenuated uncertainty aversion for more novel choices, while 8–12‐year olds’ choices were not influenced by uncertainty. For a review on reinforcement learning insights on value‐based decision making across development, see Nussenbaum and Hartley (2019). How these developmental patterns extend to economic trust behavior remains particularly unclear, given the scarcity of research in this domain.

The only computational model of the development of economic trust behavior is a study by Westhoff et al. (2020) focusing on the developmental changes from late childhood into adolescence and early adulthood. Specifically, by testing participants ages 8–23 years in a repeated Trust Game and using a reinforcement learning model that took into account decaying learning rates and fairness preferences, the authors found a distinct learning pattern in 8–11‐year‐olds: their learning rates remained high throughout 15 trials, suggesting that they kept adjusting their trust behaviors to the environments. Conversely, adolescents and young adults quickly learned about the trustworthiness of their environment and stopped updating their own decisions after a few trials. It is unclear why the youngest age group had a distinct learning pattern. One possibility is that younger children are more lenient. They might give people who betrayed their trust the benefit of the doubt, so it took them longer to label their partners as good or bad. Another possibility that causes concern is that the youngest age group might not have fully understood the game since they saw the same complicated payoff matrix as adults did. Unfortunately, the authors did not include a comprehension question to check the youngest age group's level of understanding. In fact, there is a meaningful gap in the literature that systematically examines how young children use experienced trustworthiness to inform their trust expectations and behaviors. This may be due, in part, to the methodological challenges of designing experimental paradigms concise yet clear enough to fit enough repeated trials within young children's attention span to meaningfully capture subtle behavioral change over time.

In summary, reinforcement learning modeling is gaining popularity among researchers who are interested in modeling the emergence of trust across repeated interactions. Despite the promise of this method, no studies thus far have used this approach to examine trust in young children as they are starting to engage in economic trust behaviors. Our study addresses this gap using a developmentally appropriate repeated Trust Game design that is suitable for combining behavioral analyses and computational modeling to examine how learning and trust adjustment unfold in early and middle childhood.

## 2 Current Study

The current study sought to investigate how 6–11‐year‐old children's trust behavior adapts as they learn about the trustworthiness of novel partners through experience. We used a computerized version of the Trust Game adapted from the validated in‐person apparatus used in Rosati et al. (2019). Children made economic trust decisions with two partners, one mostly trustworthy and one mostly untrustworthy, without prior knowledge of their trustworthiness. We asked: (a) are young children able to learn about their partner's trustworthiness through experience?, (b) how quickly do children adapt to interacting with trustworthy and untrustworthy partners?, and (c) does the learning process differ by age?

We chose our target age range of 6–11 years based on prior literature on children's economic trust, which showed that children as young as 6 could adapt to trustworthy and untrustworthy partners when they behaved consistently (Rosati et al. 2019). We capped our upper bound at 11 years old because we did not want to include adolescents, whose decision making differs markedly in risk and social preferences (e.g., Van Den Bos et al. 2010). Although we initially intended to include 5‐year‐olds to capture the earlier emergence of economic trust, we ultimately excluded this age group because children of this age did not consistently complete piloting sessions and had difficulty using computer keyboards during online testing.

Based on prior research, we hypothesized that children would be able to correctly identify the trustworthiness of their novel partners after playing with them. Moreover, we predicted that there would be a significant interaction between age and partner type (trustworthy vs. untrustworthy) on children's overall rate of trust as they played with both partners. Specifically, we predicted that older children would show a greater differentiation in sharing with trustworthy and untrustworthy partners.

In addition to examining overall behavioral trust patterns, we used reinforcement learning modeling to model age‐related differences in the learning mechanism and how fast children adapt their trust behavior through learning from experience. To gain further insight on the learning mechanism, we explored two extensions to the basic reinforcement learning model: (i) incorporating individual prior trust expectations as the learning prior that gets updated through experience, and (ii) allowing learning rates, or the degree to which expectations are updated after each trial, to gradually decline across trials, reflecting possible consolidation of trustworthiness impressions and decreases in behavioral flexibility over time. Thus, we fitted four variants of reinforcement learning models (with and without prior trust expectations; with and without decaying learning rates) to our experimental data for each age cohort to estimate different parameters across cohorts. Since this analysis was exploratory, we did not have specific hypotheses to which model formulation would be best fitting. This study was preregistered here: https://aspredicted.org/mpt8‐hz5g.pdf.

## 3 Method

### 3.1 Participants

Our final sample included *N* = 96 children from 6 to 11 years old (*M* = 8.89, SD = 1.65; 47 girls, 48 boys, 1 did not report. The sample size was determined based on similar developmental studies, in particular Rosati et al. (2019). Six additional children were tested but failed comprehension checks and were therefore excluded as per our preregistered exclusion criteria. In‐person testing was conducted either at local parks (*n* = 9) or at local museums (*n* = 34) in a small suburban city in the midwestern United States. For online‐testing, we recruited participants through an online database (*n* = 53) maintained by the lab. Since we did not find any significant differences in participation format or gender in overall rate of trust decisions, we collapsed the data for further analyses. For details on the effects of testing format and other demographic variables on children's economic trust, see online Supporting Information.

Legal guardians provided information about our child participants’ gender, date of birth, race and ethnicity, estimated household income, and the highest level of education received by the participant's primary caregiver. Date of birth and test date were used to calculate children's age at the time of testing. Household income and primary caregiver education were used as proxies for socioeconomic status in our exploratory analyses. Our sample was predominantly White (64%) and non‐Hispanic (85.4%). Majority of the participants come from highly educated, upper middle‐class backgrounds, with 60.4% of the primary caregivers receiving graduate degrees (master's, doctoral, or professional degree) and 56.3% of the families reported annual household income higher than $100,000. For detailed information on participant demographics, see Supporting Information. All study procedures were approved by the [blinded for review].

### 3.2 Design and Procedure

After obtaining written consent from legal guardians and verbal assent from our child participants, we introduced children to the trust game, which we called “coin game” during the testing session. Children answered a set of comprehension check questions and two questions about their baseline trust expectations. They then played a total of 40 trials of the Trust Game. We used a within‐person design such that participants played with both the trustworthy and the untrustworthy partner in 20 trials each, in a predetermined order. We determined trial order in a semi‐randomized way such that the first two trials with each partner were always congruent trials (trustworthy partner shares back fairly, untrustworthy partner takes all coins). We then determined trial numbers for the incongruent trials amongst randomized sequences so that the incongruent trials are interspersed within the 20 trials. Lastly, participants answered follow‐up questions about their post‐game trust estimates and their generalized trust.

#### 3.2.1 Task Familiarization

For in‐person testing sessions, children sat in front of a computer and used the keyboard to make decisions in the game. The experimenter sat next to the participants; parents and siblings were asked to minimize interaction with their children during the game. For online testing sessions, children and parents joined Zoom meeting rooms and shared their screen with the experimenter. Participants were introduced to the coin game, a computerized, child‐friendly repeated Trust Game adapted from the in‐person paradigm validated in Rosati et al. (2019). The game was created with Psychopy (Peirce et al. 2019) and administered through Pavlovia.

First, the experimenter introduced the coins as a valuable resource that children could accumulate throughout the game: the more coins they collected in their piggy bank, the more prizes they could earn at the end of the game. Using virtual tokens like coins as incentives in online decision‐making tasks for children is a well‐validated method that has become increasingly popular in developmental psychology studies (e.g., Corbit et al. 2023; Lee and Warneken 2022) and has been validated to yield similar results as in‐person experiments that use physical prizes as incentives (Ahl et al. 2023). Children also learned that they would be playing the coin game with other kids joining from their home, who would also earn coins throughout the game and exchange them for prizes.

**FIGURE 1:**
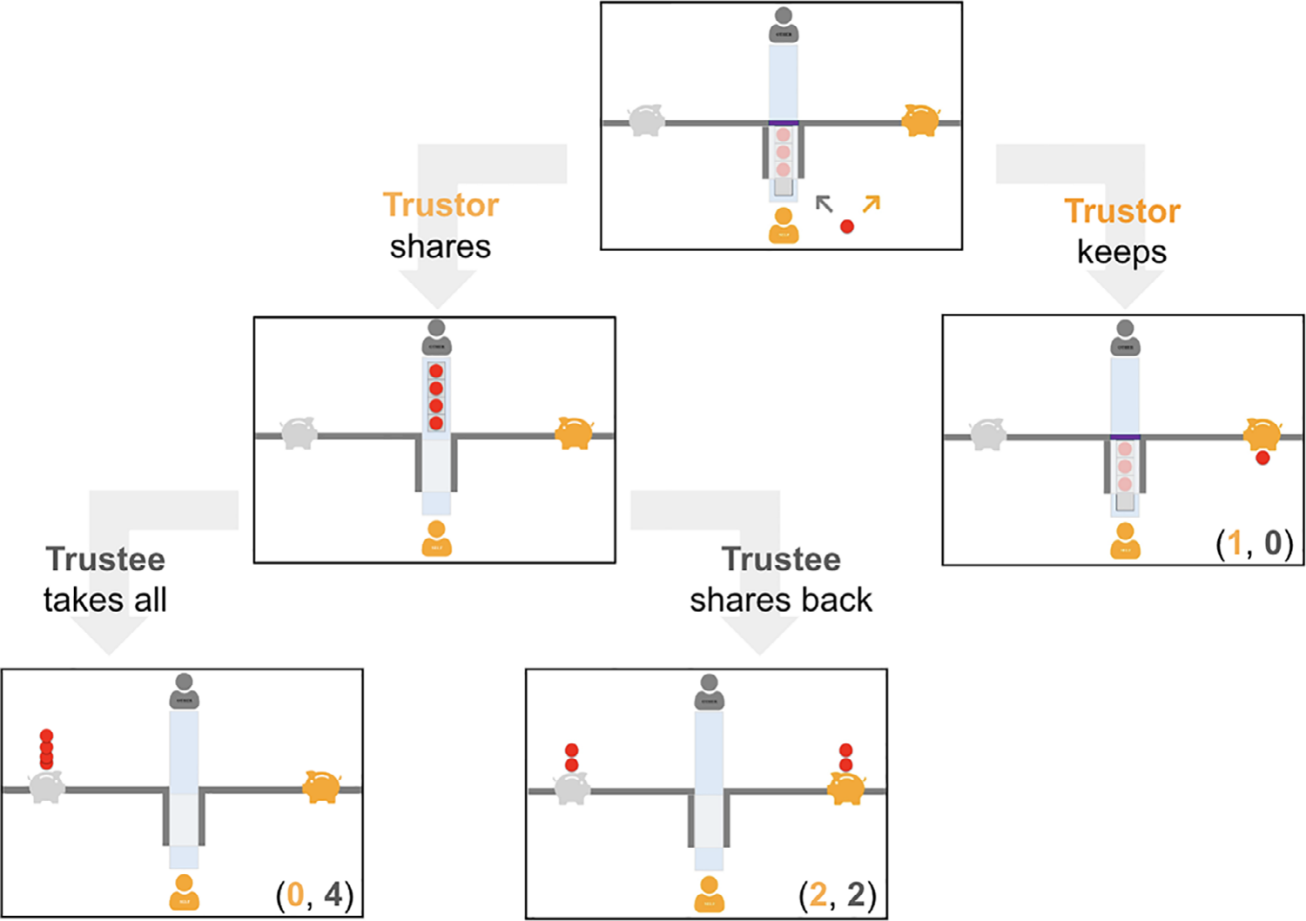
Sample stimuli in the Trust Game represented in the format of a decision tree. *Note*: Payout specified in parentheses: the first number is the number of coins the participant (trustor) earns and the second number is the number of coins the partner (trustee) earns.

Then, children learned how the middle tray with three preloaded coins work. The experimenter explained that when the blocker is in place, neither the child or their partner can have the three coins in the middle tray. The only time the blocker would come out is when the tray was full with 4 coins. Then children saw a demonstration of putting a coin onto the tray to complete it and the blocker coming out as a result. Next, children learned that once the middle tray is full, they can push it across the table to the other side, and it is up to the other player to decide how they want to divide up the 4 coins. We then presented children with two possible ways the other child could divide up the coins: one is splitting the four coins equally (2:2), and the other is taking all four coins for themselves (0:4; see Figure 1). These were also the two outcomes that we presented in the repeated game, although we did not explicitly state that.

Finally, they learned that they could also directly put the coin into their own bank and saw a demonstration of one coin going directly into their own piggy bank (1:0; see Figure 1).

##### 3.2.1.1 Comprehension Checks

Participants answered four questions about the number of tokens themselves and their partner would get in two sample stimuli and four questions about when the partner has access to coins in the middle tray. These questions were designed to ensure that children understood the mechanism of the virtual Trust Game setup.

##### 3.2.1.2 Partner Introduction

After participants passed the comprehension check questions, we introduced them to two other kids that would be playing with them. The only difference between the two players were the colors of their avatars on screen: one was a blue player and the other was a green player. To make sure that participants could distinguish between the two colors, we presented the players one at a time and asked which player they were looking at.

##### 3.2.1.3 Baseline Trust

Then, we assessed participants’ baseline trust expectations by asking them to guess how many out of the four coins each of the other players would keep when given the opportunity. We then calculated individual baseline trust expectations by subtracting the average number of tokens participants expected their trustees to keep from four. This gave us the average number of tokens participants expected to receive from their partners as reciprocation for their investment, before they could observe how many tokens the respective partners actually shared with them during the game.

To validate this design that the color or order of the partners presented on screen would not affect children's trust behavior, we used a paired t‐test to examine children's baseline trust expectations toward the two partners. We found that children did not expect the two partners to reciprocate coins differently, *t*(95) = 0.97. *p* = 0.34. On average, children expected the blue partner to keep 2.77 out of the 4 coins and the green partner to keep 2.66 out of the 4 coins they would receive. This finding also suggested that children expected their partners to reciprocate but in a slightly more self‐serving manner in this game.

Since it is crucial that participants perceive the coin game as not merely a fun game but also a reciprocal social interaction, experimenters pretended to call other experimenters to “make sure that the other kids are also ready” before the start of the games.

#### 3.2.2 Repeated Trust Game

Participants then played a total of 40 trials of the Trust game with two partners. Unbeknownst to the participants, one partner was generally trustworthy (reciprocating 80% of the trials) and the other was generally untrustworthy (reciprocating 20% of the trials). We chose the 80% versus 20% split based on prior literature, in particular Chang et al. (2010) who used an 80/20 split with adult participants, and Westhoff et al. (2020) who used a 73/27 split with participants 8–23 years of age. Since our sample is younger than those in prior studies, we chose the split with less variability and a higher trial number as a conservative initial approach. Trial order was pre‐determined and fixed across all participants so that all participants received the same learning environment. The first two trials with each partner were always congruent behaviors (trustworthy partner reciprocates, untrustworthy partner takes all). Four out of the 20 trials with each partner type were incongruent, which were intentionally interspersed throughout the trials. Children were generally able to finish the 40 trials without interruption. Their binary decisions (share or keep) on each trial were used as our key dependent variable.

#### 3.2.3 Post‐Game Questions

To assess perceived trustworthiness after the repeated Trust Game experience, we asked participants to estimate how often they thought each of the partners shared with them (1 – “none of the time” to 4 – “all of the time”) and to identify which of the two partners shared more with them. Then, we assessed children's generalized trust using the one‐item from the General Social Survey (GSS; Smith et al. 2013). Finally, participants were asked whether they believed that they were interacting with other children, for which 71 out of 96 participants included in the final sample reported that they believed they were playing with other kids (instead of playing pretend) after the game, suggesting that children were mostly convinced that they were playing the game in a social context. See Supporting Information for more details on the post‐game questionnaire.

### 3.3 Analytic Approach

We performed our data analyses using general linear mixed models and reinforcement learning models in R (version 4.5.0). For the general linear mixed models, our main dependent variable was the overall rate of trust, calculated by dividing participant's total number of trials they shared with each partner by 20. Our main independent variables were condition (trustworthy or untrustworthy) and age. Age was a continuous variable calculated by taking the difference between testing date and participant birthday. One deviation from the pre‐registered analysis is that we mean‐centered the continuous age variable due to collinearity issues. For the general linear mixed models, we started with a null model with participant ID as a random intercept and added our hypothesis‐driven independent variables and their interactions as main effects one at a time to see if they improved model fit based on likelihood ratio test result statistics (*p* values). Then, we explored the effects of demographic variables (gender, household income, and primary caregiver education level), belief that the partners were real kids, baseline trust, and generalized trust on overall trust rate.

For our computational modeling approach, we constructed reinforcement learning models following Sutton and Barto (2018) and Westhoff et al. (2020). Following the basic logic of reinforcement learning, the computational models assume that participants maximize reward on each trial by making a decision that is informed by their expectations of the partner's trustworthiness, which is learned and updated based on their past experience with the partner. Formally, on each trial (*t*), expectation (*p*) that the partner would choose to reciprocate is updated by a proportion of the prediction error (PE), estimated as the learning rate (*λ*):

$${p_{t}}_{+1}=p_{t}+\lambda \cdot PE$$

where PE is the difference between the partner's behavior on trial *t* and the expectation prior *p*. The free parameter *λ* is bounded between 0 and 1, with 0 indicating no update of *p* based on PE and 1 indicating a complete update.

The expectation *p* is therefore used to calculate the relative weights (*w*) of choosing to share (*S*) or keep (*K*). On each trial (*t*), the expected relative payoff of choosing *S* or *K* are:

$$w_{S,t}=p_{t}\cdot pay\_a+\left(1-p_{t}\right)\cdot pay\_b$$

$$w_{K,t}=p_{t}\cdot pay\_c+\left(1-p_{t}\right)\cdot pay\_d$$

where the payoff matrix defined by our design is:

pay_a=2,pay_b=0,pay_c=1,pay_d=1

Therefore, effectively,

$$w_{S,t}=2\cdot p_{t}\;\mathrm{and}\;w_{K,t}=1$$

The probability of choosing *S* is determined by a standard softmax function:


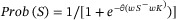




Here, *θ* represents exploration rate, or decision sensitivity, that is used to estimate the extent to which a decision maker is willing to explore alternative options rather than exploiting known best options. It helps capture the stochasticity in people's decisions that explains why people sometimes make exploratory choices instead of consistently choosing the most rewarding option. We allowed the free parameter *θ* to vary between 0 and 5 following Westhoff et al. (2020), with higher values indicating lower level of exploration and lower values indicating higher level of exploration.

To assess whether there are meaningful developmental differences in the learning mechanism between Ages 6 and 11 with adequate number of observations for our group‐level parameter estimation, we divided our sample into three age groups: 6‐ and 7‐year‐olds (*n* = 30), 8‐ and 9‐year‐olds (*n* = 38), and 10‐ and 11‐year‐olds (*n* = 28). We were interested in (1) whether allowing learning rate decline better describe trust learning and (2) whether individual baseline trust could serve as priors. Therefore, for each age group, we constructed four reinforcement learning models following this two‐by‐two design. We then used learning loss as the basis for model comparisons to select best‐fitting models for each age group.

In models that allowed for learning rate decline, we defined learning rate as

$$\lambda_{t}=\lambda_{0}\cdot i^{-\tau }$$

where *i* denotes trial number (between 1 and 20) and *τ* denotes a free parameter that reflects the speed of the decay (allowed to vary between 0 and 5). In models that incorporated individual baseline trust expectations, we allowed *p* to be the individual‐level self‐reported baseline trust expectations between 0 and 1, with 0 indicating expectation for the partner to be fully untrustworthy and 1 to be fully trustworthy. Specifically, to derive a probability prior *p* ∈ [0,1], we averaged each participant's baseline expected coin return across partners and divided by the maximum possible return (4)

$$p_{\text{prior}}=\text{Mean expected coins}/4$$

In models that did not include individual baseline trust prior, the default prior was set at *p* = 0.5 to reflect an indifference about the partner's trustworthiness.

Parameters were estimated via maximum likelihood estimation. The negative log‐likelihood (*G* ^2^ = −2LL) was minimized using the Nelder–Mead method implemented in R's optim function. The likelihood was computed by summing trial‐level log probabilities of observed choices across groups of participants, resulting in group‐level parameter estimates. To address concerns about local minima, we re‐estimated each model using 10 random starting values drawn from our defined parameter bounds and reported the solution with the lowest negative log‐likelihood. Results we report below were stable across initializations. We report parameter estimates and Hessian standard errors. For model comparison, we computed Bayesian Information Criterion (BIC) that penalizes model complexity and compared model BICs with BIC for a random choice model. Lower values indicate better fit.

## 4 Results

### 4.1 Overall Trust Rates

**FIGURE 2:**
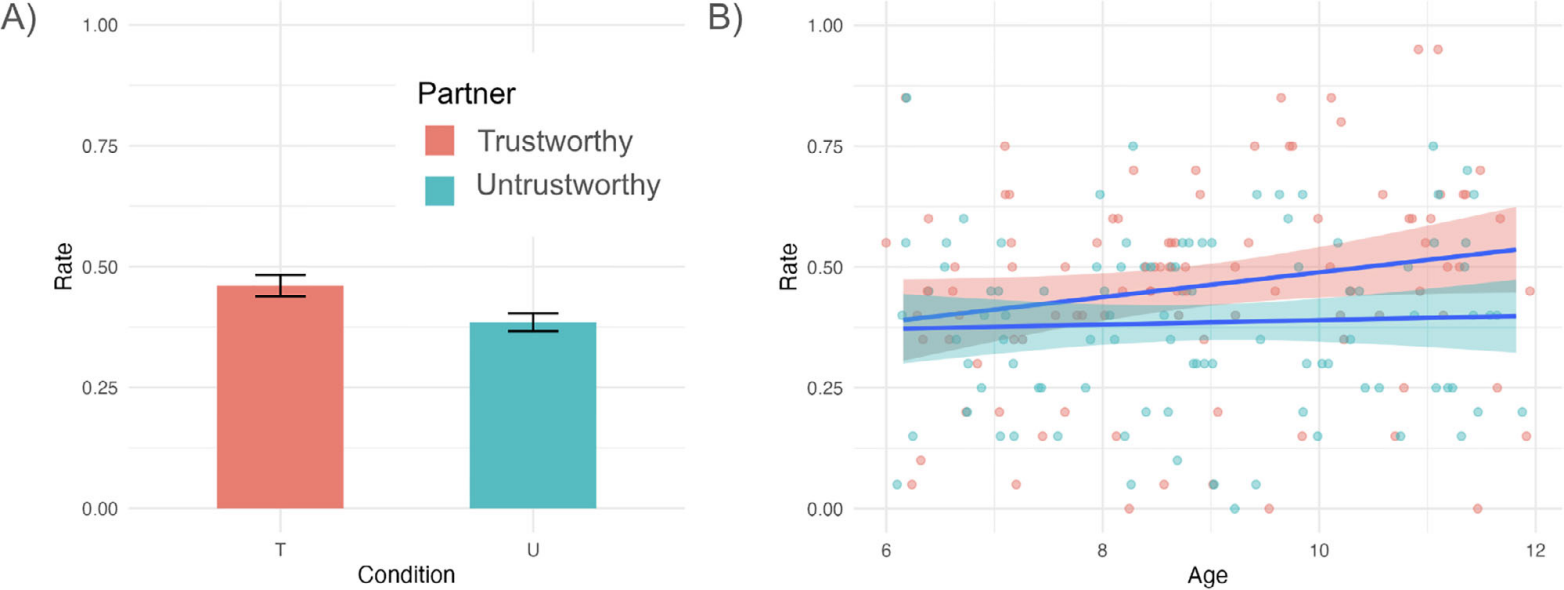
Overall trust rates by (A) partner type and (B) by age and partner type. *Note*: Trust rates represent the proportion of trials participants shared their coins with their partners. Error bars represent (A) standard errors and (B) 95% confidence intervals.

First, we examined participants’ overall rate of trust aggregated across the 20 trials they played with each partner to see if they differentiated between the two partners. Results showed that the best‐fitting model contained only a main effect of the trustworthiness condition, *β* = −0.076, SE = 0.021, *t*(95) = −3.68, *p* < 0.001, showing that participants shared more coins with the trustworthy than the untrustworthy partner (see Figure 2A and Table 1).

**TABLE 1:** The effects of condition, age, and baseline trust on trust decisions.

	Step 1	Step 2	Step 3
Intercept	0.460^***^	0.460^***^	0.356^***^
	(0.020)	(0.020)	(0.036)
Condition	−0.076^***^	−0.076^***^	−0.076^***^
	(0.021)	(0.020)	(0.020)
Age		0.026^*^	0.027^*^
		(0.012)	(0.012)
Condition × age		−0.021	−0.021
		(0.012)	(0.012)
Baseline			0.081^***^
			(0.023)
*R^2^*	0.035	0.056	0.135
AIC	−94.027	−94.972	−104.67
BIC	−80.997	−75.427	−81.863

*Notes*: Standardized estimates (beta) reported with standard errors in paratheses.

^*^ *p* < 0.05.

^**^ *p* < 0.01.

^***^ *p* < 0.001.

Our hypothesis‐driven model with an interaction between trustworthiness condition and age was not significantly better than the main effect model with condition as the only predictor (*p* = 0.084). There was a significant main effect of age (*β* = 0.026, *p* = 0.04) with older children sharing more of their resources than younger children. The interaction term between condition and age was non‐significant (*β* = −0.021, *p* = 0.092) (see Table 1 for model comparisons). Together, although model comparisons did not provide reliable evidence for an age‐by‐partner interaction, descriptively, older children tended to show higher levels of trust toward the trustworthy partner while sharing rates with the untrustworthy partner remained stable across the age range (see Figure 2B).

Focusing on children who correctly answered which partner amongst the two shared more with them at the end of the game (*n* = 60), we replicated the main findings that there was a significant main effect of trustworthiness condition (*β* = −0.126, SE = 0.028, *t*(58) = −4.47, *p* < 0.001) with a larger differentiation between the two partner types, but no effects of age (*p* = 0.18) nor an interaction between condition and age (*p* = 0.41). For details on the associations between post‐hoc identification and children's trust decisions, see Supporting Information.

As a next step, we assessed whether baseline trust expectations were related to children's sharing with the trustworthy and untrustworthy partner. Using a model with participant ID as random intercepts, trustworthiness condition and age interaction and baseline trust expectations as main effects, we found a significant main effect of baseline trust expectations (*β* = 0.08, SE = 0.023, *t*(93) = 3.47, *p* < 0.001) beyond the main effects of trustworthiness condition and age, such that participants with high baseline trust expectations shared more often with their trustees, regardless of their trustworthiness or age (see Table 1 for model comparisons).

### 4.2 Trust Rates by Trial

**FIGURE 3:**
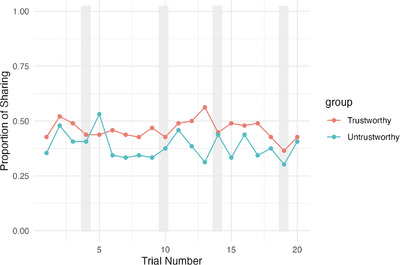
Rate of trust toward each partner by trial. *Note*: Average proportion of all children’ sharing toward the trustworthy and the untrustworthy partner over the course of 20 trials of repeated Trust Game. Gray bars indicate trials immediately after incongruent trials.

We further investigated participants’ trust decisions by trial to examine how their trust behaviors changed over the course of the game. Going beyond our preregistered analyses, we conducted exploratory analysis using a trial‐level generalized linear mixed‐effects model predicting trust decisions from partner trustworthiness, trial, age, and their interactions, with random intercepts for participants and random slopes for partner type. As seen in Figure 3, participants of all age groups did not demonstrate increasing differentiation in how they interacted with the trustworthy versus the untrustworthy partner, *p* = 0.50. Contrary to our hypothesis, age was not significantly associated with the extent to which children become more differentiating over trials to share with the trustworthy over the untrustworthy partner, *p* = 0.28, although a main effect of age remained where older children in our sample were overall more trusting. For more details on trust decisions by trial broken down in age groups, see Figure S1 in the online Supporting Information.

Although regression analyses of decision data are suitable for revealing general behavior patterns, they are not sensitive enough to capture psychological mechanisms behind how children learn from experience and adjust their own trust behaviors in a trial‐by‐trial manner. Moreover, they assume that behavioral adjustment happens in a linear fashion without being able to account for the incongruent trials included in our procedure. In fact, the behavioral fluctuation we see from Figure 3 could be the result of children's strategic exploration under this uncertain environment, which can be modeled under a reinforcement learning framework. In other words, children might have been highly flexible in their trust decisions in order to explore whether their working impressions of their partners can be updated with new experience. These strategic motivations cannot be simply captured with the regression approach. Therefore, we explored by trial data more using reinforcement learning modeling in the section below.

### 4.3 Reinforcement Learning Models

**FIGURE 4:**
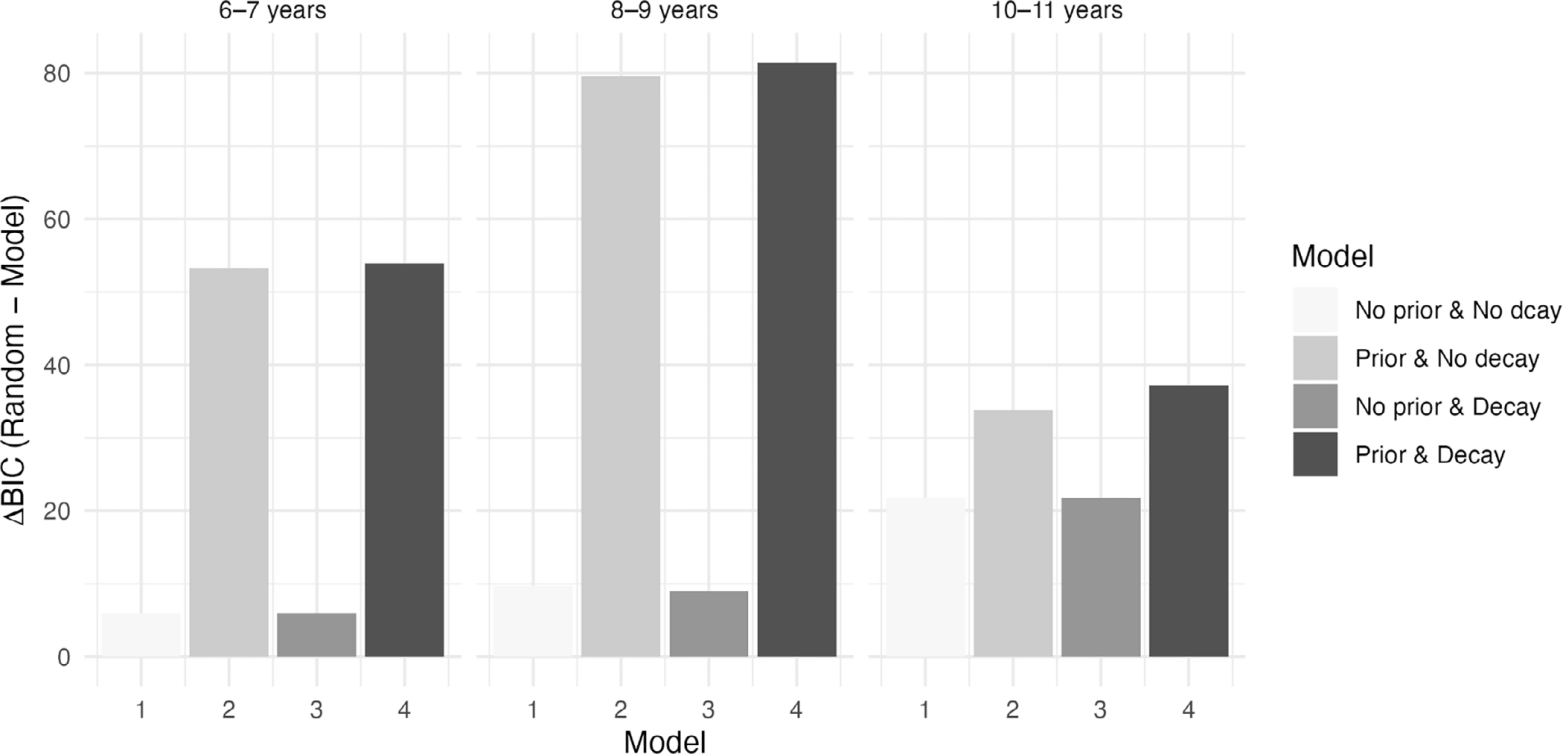
Reinforcement learning model fit indices (Bayesian Information Criterion [BIC]) by age groups. *Note*: BIC differences between the four possible models and a random‐choice model for 6–7‐, 8–9‐, and 10–11‐year‐olds. As lower BIC indicates better model fit, larger BIC differences between random model and hypothesis‐driven model indicates better model fit.

To understand developmental differences in how children update their expectations about their partners’ trustworthiness and adjust their own behaviors accordingly, we used reinforcement learning models to examine their behaviors. Specifically, we were interested in quantifying children's learning rate, decline in learning, and to assess whether prior trust expectations further predicted children's behavior. To explore the developmental differences in learning and trust behaviors while maintaining reasonable sample sizes in each age group for group‐level parameter estimation, we conducted these analyses for three age groups. Based on BICs, the best‐fitting model for all three age groups was the model that included individual baseline trust expectations as priors and allowed for decline in learning rate (see Figure 4). This corroborated our findings from the general linear mixed models that individual baseline trust expectations were strongly associated with participant's decisions.

A close examination of the learning rates across age groups reveals a clear developmental pattern: the oldest age group showed the highest initial learning rate (*λ* = 0.31, SE = 0.05) with a steep decay (decay parameter = 4.99, SE = 6.37e‐06), while the youngest age group showed the lowest initial learning rate (*λ* = 0.07, SE = 0.05) with the slowest decay (decay parameter = 0.97, *S*E = 0.86). In other words, 10‐ and 11‐year‐olds engaged in faster updating during the initial trials followed by a steeper decline over subsequent trials, while 6‐ and 7‐year‐olds were slower to adjust their beliefs initially but sustained their learning over longer periods of time. The middle age group, 8‐ and 9‐year‐olds, fell in the middle for their initial learning rate (*λ* = 0.12, SE = 0.03) and showed a similar speed of learning rate decline as the oldest age group (decay parameter = 4.99, SE = 6.39e‐06). See Table S2 in the online Supporting Information for more details on the model estimates.

**FIGURE 5:**
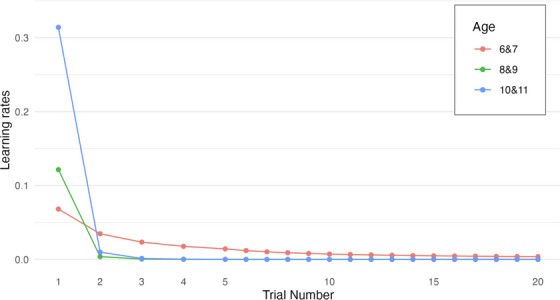
Estimated best‐fitting learning rates by age group over 20 trials. *Note*: Model‐fitted learning rate estimates for 6–7‐, 8–9‐, and 10–11‐year‐olds over 20 trials of the repeated trust game.

As shown in Figure 5, for all three age groups, learning rate estimates rapidly declined. It is worth noting that the near‐zero learning rates after Trial 5 does not necessarily suggest at‐ceiling performance with the task, as children were not consistently selectively trusting the trustworthy partner (see Figure 3). Instead, the low learning rates suggest that children's economic trust behaviors became less responsive to outcomes that violate their impressions of the two partners after Trial 5. These age‐related differences in learning rate estimates complement our regression findings in that older children in our sample were more accurate at detecting their partners’ trustworthiness.

### 4.4 Post‐Game Trust Impressions

**FIGURE 6:**
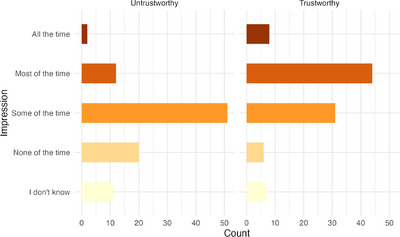
Children's self‐reported post‐game trust impressions of the two players. *Note*: In reality, the untrustworthy player shared 20% of the time while the trustworthy player shared 80% of the time.

To rule out the possibility that children were simply confused and choosing at random in the game, we examined how much children explicitly learned about the two partners after playing with them. Overall, 60 out of 96 children correctly identified the trustworthy partner. Older children were more likely to correctly identify the trustworthy partner, *χ* ^2^(1) = 8.26, *p* = 0.004, OR = 1.72, 95% CI [1.19, 2.48]. When asked to estimate how often the untrustworthy partner shared back, the majority of children reported “some of the time”. On the contrary, when asked about the trustworthy partner, children's impressions leaned more positive, with the most frequent answer being “most of the time” (see Figure 6). Although the actual frequencies of partner's sharing decision that children would be able to observe depended on how often they shared their coins with each partner, it is true that at least by design, the untrustworthy (blue) partner shared “some of the time” and the trustworthy (green) partner shared “most of the time”, suggesting that children had good estimates of their partner's behavioral pattern post‐game.

## 5 Discussion

Our primary goal was to examine developmental differences in children's economic trust when they interact with trustworthy and untrustworthy partners over repeated experience. We used both linear regression analyses and computational modeling to provide a fine‐grained mechanistic account of children's behavioral patterns. Our results showed that, overall, children trusted the trustworthy partner significantly more than the untrustworthy one, suggesting that even without prior information, and despite variability in partner behavior, children were able to infer partner trustworthiness from experience alone. Older children were more trusting toward both partners, although not significantly more selective in their trust like we hypothesized. Moreover, our reinforcement learning models revealed additional insights in the developmental differences in the learning process. The best‐fitting model suggested that children incorporated their baseline expectations of others’ trustworthiness when they interacted with novel partners and subsequently used experienced trustworthiness to update their initial expectations. Learning from those immediate experiences decreased quickly over time, possibly as children's impressions of others’ trustworthiness solidified. In other words, children's economic trust behaviors became less responsive to new outcomes over time. A clear developmental trend in learning trajectories also emerged, with 10‐ and 11‐year‐olds showing the highest initial learning rate, followed by steep decline in learning on subsequent trials, while 6‐ and 7‐year‐olds starting with the lowest learning rate with less but still substantial decline.

The current study contributes to the broader literature on children's economic trust development and decision‐making in repeated social contexts in several ways. First, it is non‐trivial that 6‐ to 11‐year‐old children showed high comprehension and satisfactory learning in a cognitively demanding economic trust task that we specifically designed to emulate important features of real‐world social environments, where individuals may behave unpredictably and trust must be built over time through experiences. For these reasons, our task meaningfully differs in important respects from prior experimental tasks on economic trust in young children. Specifically, in contrast to the procedure from Rosati et al. (2019), where children already had some prior information about the partners’ levels of trustworthiness before deciding whether to invest their own resources, our participants had to find out about their partners’ trustworthiness levels during the game itself. Moreover, in our design, the partner's responses were probabilistic rather than perfectly consistent, introducing greater uncertainty about the partners’ future behaviors and the child's adequate behavioral response. This paradigm that incorporates variability captures something important about the inferential challenge children face when navigating actual social relationships, which might explain why children's differentiation between partners emerged less clearly than in prior, more structured tasks. Together, these findings suggest that while basic mechanisms of social learning are present by early childhood, the ability to efficiently learn from ambiguous or stochastic social feedback continues to develop throughout middle childhood.

The developmental pattern we observed resembles findings from the epistemic trust development literature, though the two domains differ in ways that likely account for the difference in developmental timing and trajectories that we observed. Epistemic trust, or one's willingness to deem information provided by an informant to be accurate, relevant, and reliable (McCraw 2015; for a review, see Harris et al. 2018), develops early. By Age 3, children use an informant's past accuracy as evidence to decide who they want to trust in the future (Koenig and Harris 2005). However, these young children find it hard to reject misleading information, even when the informant has explicit malicious intent to lie to the child (Heyman et al. 2013). Starting at 4 years of age, children were able to make predictions about informants’ future accuracy based on their past accuracy and adjust their own epistemic trust based on the informant's ongoing accuracy (Clement et al. 2004; Koenig and Harris 2005; Liu et al. 2013; Ronfard and Lane 2018). Crucially, economic trust differs from epistemic trust on several dimensions including the type of risk that's involved and the observability of feedback. The risk for economic trust is more tangible, as children must decide whether to invest real resources with uncertain returns rather than to learn from unreliable information sources (Berg et al. 1995; Camerer 2003). Feedback for economic trust is also immediate, as in the current context, noisier as compared to those in classic epistemic trust paradigms (e.g., Koenig et al. 2004). These differences in demands may explain why the ability to learn selectively from social feedback continues to develop throughout middle childhood in the economic trust domain, whereas selective epistemic trust emerged earlier.

Second, we found a developmental asymmetry in how children adjusted their own trust behavior toward trustworthy and untrustworthy partners. Trust toward the trustworthy partner increased with age, whereas trust toward the untrustworthy partner remained relatively stable across the age range. This asymmetry mirrors findings from Westhoff et al. (2020), who found that adjusting to trustworthy environments increased rapidly in early to mid‐adolescence while adjusting to untrustworthy partners only improved slightly across adolescence. Our findings extend this pattern to a younger population, suggesting that the ability to selectively increase economic trust in reliable partners may develop earlier than the ability to reduce trust in unreliable ones.

Third, our finding on limited behavioral differentiation over time is particularly interesting given children's relatively accurate self‐reported impressions of their partner's trustworthiness after the game, especially amongst older children. This gap between explicit judgments and behavioral adjustment suggests that while children may form accurate impressions based on observed behavioral patterns, translating those impressions into adaptive trust behaviors is a more complex process. One possibility is that children feel the need to accrue more information before they solidify their trust decisions, especially in this context where they are interacting with anonymous peers online. In other words, children's already extensive exploration might have been extended under uncertainty (Gopnik 2020). This possibility may be especially true for younger children, who may be more used to relying on concrete social cues such as facial expressions or essentialized descriptions when making trust decisions.

Last but not least, our study demonstrated that reinforcement learning models can effectively capture children's economic trust learning dynamics in children as young as 6 years of age. This is an important addition to previous computational modeling work focused on adolescents and adults. Our modeling revealed that children engage in updating early in the interaction, but once they form a working model of a partner's trustworthiness, new information is integrated less readily, a pattern consistent with theories of confirmation bias or impression consolidation in social cognition (e.g., Darley and Gross 1983). Importantly, these learning dynamics, especially the reduced sensitivity to new information over time, closely resemble the developmental trends reported by Westhoff et al. (2020) for adolescents. We were able to extend these findings into a younger age range by using a developmentally appropriate, engaging version of a repeated trust game. This new method revealed that even 6–7‐year‐old children demonstrate reduced learning over time, expanding our understanding of when and how children begin to learn about social partners’ trustworthiness within a reinforcement learning framework.

In summary, our findings contribute to research on children's economic trust behaviors by showing that children as young as 6 years of age use experienced trustworthiness to update their prior expectations of novel social partners and guide their trust behaviors. Unlike prior studies that provided partner information or history, children in our study relied on their own exploration and experience. Computational modeling further expanded our understanding of the developmental differences in this learning process. Taken together, our findings highlight that although all age groups engaged in experience‐based learning, the efficiency with which they integrated new experience to inform their own behaviors differed systematically with age.

### 5.1 Limitations

Although the current study provides valuable insights into how children learn about trustworthiness through experience, several limitations should be considered when interpreting the findings. First, we cannot be certain whether all participants had the goal to maximize reward, as there might be heterogeneity in motivations when children played the repeated Trust Game. This question is particularly relevant given that reinforcement learning models assume agents are reward‐maximizing learners who update their behaviors based on feedback to optimize reward in a given environment. Although individual differences might exist, the overall pattern is consistent with children's attempt to maximize rewards in the task by sharing more with trustworthy over untrustworthy partners. Moreover, a large number of studies have used the similar rewards in in‐person and online studies showing that children are motivated to obtain tokens they can exchange for prizes (e.g., Ahl et al. 2023; Corbit et al. 2023; Lee and Warneken 2022). Second, the cognitive demands of the task may have differentially impacted children's performance. Successfully maximizing rewards required participants to track and integrate information about two partners’ trustworthiness across 40 trials, which puts substantial demands on working memory, sustained attention, and motivation. As such, individual and developmental differences in cognitive capacity or engagement may have influenced children's trust decisions, particularly among younger participants. Third, it is worth noting that a significant portion of our sample came from highly educated, high‐income family backgrounds and therefore is not representative of the national population. Therefore, we need to be careful in generalizing these findings beyond this demographic.

Fourth, the trial orders were predetermined with two congruent trials first and were fixed across participants, a design choice that increases experimental control but may limit ecological validity in terms of stochastic trust interactions in real life. Because outcomes were observed only after trust decisions were made and incongruent outcomes were distributed irregularly across the 20 trials, there was no predictable trial structure for children to observe. Nonetheless, using a fixed sequence may introduce anchoring effects. Our reinforcement learning approach is well suited to capturing belief updating under such structured contingencies over time, but we acknowledge that complementary event‐aligned regression analyses may be informative in less constrained designs. Sensitivity analyses excluding the first two trials yielded comparable results (see Supporting Information). Future work could extend this approach using fully randomized sequences to examine learning under greater environmental uncertainty.

Lastly, our sample was tested both in‐person and online, with unequal distribution of participants across the testing formats. Although testing formats did not make a statistically significant difference in participants' economic trust behaviors or their task comprehension, we acknowledge that the testing environment differs in social presence and potentially how the task is perceived by the participants.

### 5.2 Future Directions

Building on the current findings, future research can further investigate the developmental, contextual, and psychological determinants of children's economic trust. First, future work can consider incorporating measures of risk preferences and cognitive abilities to further explain individual differences in trust learning. In our study, there were substantial individual differences in both children's trust expectations and their trust behaviors that went beyond age‐related differences. Indeed, children with higher risk tolerance may be more willing to continue trusting after a betrayal; those with stronger working memory or cognitive flexibility may be better at tracking patterns across trials. Incorporating direct measures of these cognitive abilities could help clarify how individual variability interacts with age to affect learning patterns.

Second, differences in exploration versus exploitation trade‐offs (Gopnik 2020) could account for developmental changes and interindividual variation. Compared to adolescents or adults, children have been found to be more exploratory when faced with uncertainty in rewards (Nussenbaum et al. 2023). Future studies could vary the level of uncertainty to add to our understanding of how exploration changes with age when children engage in trust interactions with novel partners.

Third, future work can investigate the flip side of trusting: how children's own trustworthiness changes over time as they play as trustees in the repeated Trust Game. Prior work has suggested that young children reciprocate differently when they were entrusted as compared to when they were given resources out of generosity (e.g., Amir et al. 2021). It raises the question of whether children would also adjust how they choose to reciprocate, based on whether they are consistently trusted or not. Particularly, would children share back fairly to signal their trustworthiness or withhold resources to punish untrusting partners?

Finally, the current study focused on trust interactions in an online, anonymous social context. Although many of today's social interactions happen in this context, future research could investigate how learning generalizes or differs in other contexts, for example, if children were to interact with familiar partners, artificial agents, or play non‐social games. Comparing trust learning across these settings using reinforcement learning models could provide insight into how prior expectations are updated, how efficiently children learn, and how flexibly they explore across different environments.

## 6 Conclusion

Our study sheds light on children's emerging ability to flexibly adjust their economic trust behavior based on their experience interacting with novel partners. We found that older children's trust toward the trustworthy partner increased over the course of the repeated trust game, while trust toward the untrustworthy partner remained constant across the age range. We also showed the utility in adopting computational modeling methods like reinforcement learning modeling in studying social decision making in a developmental sample. Our models revealed important learning parameters, like priors, learning rates, and learning rate decline that accounted for behavioral and developmental patterns that are otherwise hidden in regression analyses. Future research should capitalize on this method to further investigate the specific mechanisms that drive children's trust behaviors as they navigate different social environments.

## Author Contributions

**Yiyan Rose Wang**: conceptualization, methodology, data curation, investigation, formal analysis, validation, visualization, writing – original draft. **Felix Warneken**: conceptualization, methodology, supervision, funding acquisition, project administration, resources, writing – review and editing.

## Artificial Intelligence Use Disclosure

Artificial intelligence tool (Claude Sonnet 4.6 by Anthropic) was used to debug reinforcement modeling codes to incorporate standard error estimates and random initial values for parameter estimation. The AI‐generated codes were reviewed and validated for accuracy. The influence of AI use on our conclusions was minimal, as these additional modeling robustness checks did not change the main findings, nor our interpretation of the findings.

## Conflicts of Interest

The authors declare no conflicts of interest.

## Supporting information




**Supporting File 1**: desc70205‐sup‐0001‐SuppMat.docx

**Supporting File 2**: desc70205‐sup‐0002‐tableS1‐S3.docx

## Data Availability

All data and analyses codes for this study are openly available on Open Science Framework, at https://osf.io/jk85q/overview?view_only=1f2ca5d198524fb9a4063ab2904b52ad.

## References

[desc70205-bib-0001] Ahl, R. E. , K. Hannan , D. Amir , A. Baker , M. Sheskin , and K. McAuliffe . 2023. “Tokens of Virtue: Replicating Incentivized Measures of Children's Prosocial Behavior With Online Methods and Virtual Resources.” Cognitive Development 66: 101313. 10.1016/j.cogdev.2023.101313.

[desc70205-bib-0002] Amir, D. , W. S. Parsons , R. E. Ahl , and K. McAuliffe . 2021. “Trustworthiness Is Distinct From Generosity in Children.” Developmental Psychology 57, no. 8: 1318–1324. 10.1037/dev0001214. 34591574

[desc70205-bib-0003] Balliet, D. , and P. A. M. Van Lange . 2013. “Trust, Conflict, and Cooperation: A Meta‐Analysis.” Psychological Bulletin 139, no. 5: 1090–1112. 10.1037/a0030939. 23231532

[desc70205-bib-0004] Berg, J. , J. Dickhaut , and K. McCabe . 1995. “Trust, Reciprocity, and Social History.” Games and Economic Behavior 10, no. 1: 122–142. 10.1006/game.1995.1027.

[desc70205-bib-0005] Bó, P. D. 2005. “Cooperation Under the Shadow of the Future: Experimental Evidence From Infinitely Repeated Games.” American Economic Review 95, no. 5: 1591–1604. 10.1257/000282805775014434.

[desc70205-bib-0038] Camerer, C. F. 2003. Behavioral Game Theory: Experiments in Strategic Interaction. Russell Sage Foundation.

[desc70205-bib-0006] Chang, L. J. , B. B. Doll , M. van't Wout , M. J. Frank , and A. G. Sanfey . 2010. “Seeing Is Believing: Trustworthiness as a Dynamic Belief.” Cognitive Psychology 61, no. 2: 87–105. 10.1016/j.cogpsych.2010.03.001. 20553763

[desc70205-bib-0007] Clement, F. , M. Koenig , and P. Harris . 2004. “The Ontogenesis of Trust.” Mind and Language 19, no. 4: 360–379. 10.1111/j.0268-1064.2004.00263.x.

[desc70205-bib-0008] Corbit, J. , M. Dockrill , S. Hartlin , and C. Moore . 2023. “Intuitive Cooperators: Time Pressure Increases Children's Cooperative Decisions in a Modified Public Goods Game.” Developmental Science 26, no. 4: e13344. 10.1111/desc.13344. 36399363

[desc70205-bib-0009] Darley, J. M. , and P. H. Gross . 1983. “A Hypothesis‐Confirming Bias in Labeling Effects.” Journal of Personality and Social Psychology 44, no. 1: 20–33. 10.1037/0022-3514.44.1.20.

[desc70205-bib-0010] Engle‐Warnick, J. , and R. L. Slonim . 2006. “Learning to Trust in Indefinitely Repeated Games.” Games and Economic Behavior 54, no. 1: 95–114. 10.1016/j.geb.2004.10.009.

[desc70205-bib-0011] Evans, A. M. , U. Athenstaedt , and J. I. Krueger . 2013. “The Development of Trust and Altruism During Childhood.” Journal of Economic Psychology 36: 82–95. 10.1016/j.joep.2013.02.010.

[desc70205-bib-0012] Fehr, E. 2009. “On the Economics and Biology of Trust.” Journal of the European Economic Association 7, no. 2–3: 235–266. 10.1162/JEEA.2009.7.2-3.235.

[desc70205-bib-0013] Fetchenhauer, D. , and D. Dunning . 2012. “Betrayal Aversion Versus Principled Trustfulness—How to Explain Risk Avoidance and Risky Choices in Trust Games.” Journal of Economic Behavior & Organization 81, no. 2: 534–541. 10.1016/j.jebo.2011.07.017.

[desc70205-bib-0014] Gopnik, A. 2020. “Childhood as a Solution to Explore–Exploit Tensions.” Philosophical Transactions of the Royal Society B: Biological Sciences 375, no. 1803: 20190502. 10.1098/rstb.2019.0502. PMC7293160 32475327

[desc70205-bib-0015] Koenig, M. A. , F. Clément , and P. L. Harris . 2004. “Trust in Testimony: Children's Use of True and False Statements.” Psychological Science 15, no. 10: 694–698. 10.1111/j.0956-7976.2004.00742.x. 15447641

[desc70205-bib-0016] Harris, P. L. , M. A. Koenig , K. H. Corriveau , and V. K. Jaswal . 2018. “Cognitive Foundations of Learning From Testimony.” Annual Review of Psychology 69, no. 1: 251–273. 10.1146/annurev-psych-122216-011710. 28793811

[desc70205-bib-0017] Heyman, G. D. , L. Sritanyaratana , and K. E. Vanderbilt . 2013. “Young Children's Trust in Overtly Misleading Advice.” Cognitive Science 37, no. 4: 646–667. 10.1111/cogs.12020. 23294130 PMC8063353

[desc70205-bib-0018] Johnson, N. D. , and A. A. Mislin . 2011. “Trust Games: A Meta‐Analysis.” Journal of Economic Psychology 32, no. 5: 865–889. 10.1016/j.joep.2011.05.007.

[desc70205-bib-0019] Koenig, M. A. , and P. L. Harris . 2005. “Preschoolers Mistrust Ignorant and Inaccurate Speakers.” Child Development 76, no. 6: 1261–1277. 10.1111/j.1467-8624.2005.00849.x. 16274439

[desc70205-bib-0020] Lee, Y. , and F. Warneken . 2022. “The Influence of Age and Experience of (Un)Fairness on Third‐Party Punishment in Children.” Social Development 31, no. 4: 1176–1193. 10.1111/sode.12604.

[desc70205-bib-0021] Liu, D. , K. E. Vanderbilt , and G. D. Heyman . 2013. “Selective Trust: Children's Use of Intention and Outcome of Past Testimony.” Developmental Psychology 49, no. 3: 439–445. 10.1037/a0031615. 23339589

[desc70205-bib-0022] Margoni, F. , E. Nava , and L. Surian . 2022. “Do Children Selectively Trust Leaders and Prosocial Agents in an Economic Exchange?” Developmental Psychology 58, no. 1: 152–160. 10.1037/dev0001282. 34928628

[desc70205-bib-0023] McCraw, B. W. 2015. “The Nature of Epistemic Trust.” Social Epistemology 29, no. 4: 413–430. 10.1080/02691728.2014.971907.

[desc70205-bib-0024] Nussenbaum, K. , and C. A. Hartley . 2019. “Reinforcement Learning Across Development: What Insights Can We Draw From a Decade of Research?” Developmental Cognitive Neuroscience 40: 100733. 10.1016/j.dcn.2019.100733. 31770715 PMC6974916

[desc70205-bib-0025] Nussenbaum, K. , R. E. Martin , S. Maulhardt , et al. 2023. “Novelty and Uncertainty Differentially Drive Exploration Across Development.” eLife 12: e84260. 10.7554/eLife.84260. 37585251 PMC10431916

[desc70205-bib-0026] Peirce, J. , J. R. Gray , S. Simpson , et al. 2019. “PsychoPy2: Experiments in Behavior Made Easy.” Behavior Research Methods 51, no. 1: 195–203. 10.3758/s13428-018-01193-y. 30734206 PMC6420413

[desc70205-bib-0027] Ronfard, S. , and J. D. Lane . 2018. “Preschoolers Continually Adjust Their Epistemic Trust Based on an Informant's Ongoing Accuracy.” Child Development 89, no. 2: 414–429. 10.1111/cdev.12720. 28105637

[desc70205-bib-0028] Rosati, A. G. , N. Benjamin , K. Pieloch , and F. Warneken . 2019. “Economic Trust in Young Children.” Proceedings of the Royal Society B: Biological Sciences 286, no. 1907: 20190822. 10.1098/rspb.2019.0822. PMC6661339 31337306

[desc70205-bib-0029] Rousseau, D. M. , S. B. Sitkin , R. S. Burt , and C. Camerer . 1998. “Introduction to Special Topic Forum: Not so Different After All: A Cross‐Discipline View of Trust.” The Academy of Management Review 23, no. 3: 393–404.

[desc70205-bib-0031] Siddique, S. , L. Jeffery , R. Palermo , J. R. Collova , and C. A. M. Sutherland . 2022. “Children's Dynamic Use of Face‐ and Behavior‐Based Cues in an Economic Trust Game.” Developmental Psychology 58, no. 12: 2275–2286. 10.1037/dev0001438. 36136782

[desc70205-bib-0032] Smith, T. W. , P. V. Marsden , and M. Hout . 2013. General Social Survey, 1972–2010 [Cumulative File]. Roper Center for Public Opinion Research, University of Connecticut [distributor], Inter‐university Consortium for Political and Social Research [distributor]. 10.3886/ICPSR31521.v1.

[desc70205-bib-0033] Sutton, R. S. , and A. G. Barto . 1998. Reinforcement Learning: An Introduction. The MIT Press.

[desc70205-bib-0034] Sutton, R. S. , and A. G. Barto . 2018. Reinforcement Learning: An Introduction (2nd ed.). The MIT Press.

[desc70205-bib-0035] Van Den Bos, W. , M. Westenberg , E. Van Dijk , and E. A. Crone . 2010. “Development of Trust and Reciprocity in Adolescence.” Cognitive Development 25, no. 1: 90–102. 10.1016/j.cogdev.2009.07.004.

[desc70205-bib-0036] Warneken, F. 2018. “How Children Solve the Two Challenges of Cooperation.” Annual Review of Psychology 69, no. 1: 205–229. 10.1146/annurev-psych-122216-011813. 28876999

[desc70205-bib-0037] Westhoff, B. , L. Molleman , E. Viding , W. van den Bos , and A. C. K. van Duijvenvoorde . 2020. “Developmental Asymmetries in Learning to Adjust to Cooperative and Uncooperative Environments.” Scientific Reports 10, no. 1: 21761. 10.1038/s41598-020-78546-1. 33303840 PMC7729944

