# Supplementary material for: Trusting Wisely? Developmental Changes in How Children Learn and Adapt to Partner Trustworthiness
Source: Dev Sci. 2026 Apr 26;29:e70205. doi: 10.1111/desc.70205 (PMC13111782; doi:10.1111/desc.70205)

Supplementary Materials for

**Trusting wisely? Developmental changes in how children learn and adapt to partner trustworthiness**

[Author information blinded for review]

##

## Post-game Questionnaire

**Post-game trustworthiness impressions**

1. How often do you think the [blue/green] player shared with you?
   1. All of the time
   2. Most of the time
   3. Some of the time
   4. None of the time
   5. I don’t know
2. Who do you think shared more with you? The green player or the blue player?
   1. Green player
   2. Blue player
   3. About the same

**Generalized trust**

1. Generally speaking, would you say that most people can be trusted or that you should always be careful trusting people, or does it depend?
   1. Can be trusted
   2. It depends
   3. Should be careful
   4. I don’t know

**Human Player**

1. Did you think that you were playing with other kids or do you think it might have been playing pretend?
   1. Real b. Pretend

## Table S1. Participant demographic information

| **Variable** | **Categories** | **Frequency** | **Percentage** |
| --- | --- | --- | --- |
| Gender |  |  |  |
|  | Girl | 47 | 48.96% |
|  | Boy | 48 | 50% |
|  | Did not report | 1 | 1.04% |
| Race |  |  |  |
|  | Asian or Asian American | 10 | 10.41% |
|  | Black or African American | 9 | 9.38% |
|  | Multiracial | 11 | 11.46% |
|  | Native American or Pacific Islander | 1 | 1.04% |
|  | White or European American | 62 | 64.58% |
|  | Other | 3 | 3.13% |
| Ethnicity |  |  |  |
|  | Hispanic or Latino | 14 | 14.58% |
|  | Not Hispanic or Latino | 82 | 85.42% |
| Household Income |  |  |  |
|  | Up to $39,999 | 6 | 6.25% |
|  | $40,000 to $69,999 | 18 | 18.75% |
|  | $70,000 to $99,999 | 12 | 12.5% |
|  | $100,000 to $149,999 | 20 | 20.83% |
|  | $150,000 to $ 199,999 | 15 | 15.63% |
|  | $200,000 or more | 19 | 19.79% |
|  | Prefer not to say | 6 | 6.25% |
| Caregiver Education |  |  |  |
|  | Some high school or less | 0 | 0% |
|  | Highschool | 4 | 4.17% |
|  | Some College (1-3 years, Associate’s degree) | 9 | 9.38% |
|  | 4 year degree (Bachelor’s degree) | 25 | 26.04% |
|  | Graduate degree (Master’s, doctoral, or professional degree) | 58 | 60.42% |
| Testing location |  |  |  |
|  | Local parks | 9 | 9.38% |
|  | Local museums | 34 | 35.43% |
|  | Online | 53 | 55.21% |

## Exploratory results for the effects of demographic background on children’s trust decisions

We explored whether other demographic characteristics of children related to children’s trust decisions in the repeated Trust Game. Based on the condition and age interaction model we reported in the main text, we added demographic variables one at a time to see if they improved overall model fit. Gender was not associated with overall trust, *p* = .47. Neither primary caregiver education level nor household income were associated with overall trust, *p*s > .59. We did not run this analysis on race and ethnicity because we had little reason to believe that these variables would be associated with children’s trust behaviors in the study and that some group sizes would be really small if we were to run the analysis.

##

## Exploratory results for the effects of human player belief on children’s trust decisions

We also explored whether children’s belief that they’re playing with real human players would have an effect on their trust behaviors. Overall, 71 participants believed that they played with other kids, 23 reported thinking that it was just playing pretend, and 2 participants chose not to answer. It is worth noting that simply by asking this question, we could have induced suspicion that the partners in the game were not real human players, so this measure needs to be considered with a grain of salt. Based on the condition and age interaction model we reported in the main text, we added the human player belief variable to see if it improved overall model fit. We found that human player belief was not significantly associated with children’s trust decisions, *p* = .39, suggesting that whether children believed they were really playing with other kids or just playing pretend did not have an effect on how likely they were to trust the other player.

##

## Exploratory results for the effects of generalized trust on children’s trust decisions

We examined children’s self-reported generalized trust and explored whether it related to children’s trust decisions when they were playing the game. Very few children in our sample reported “most people can be trusted” (5 out of 96) Another 33 children reported “always be careful”. The majority of children (50 out of 96) said that “it depends” and some spontaneously proceeded to give reasoning to why some people can be trusted while others cannot. There was not much age-related variation in how children responded to this question, although younger children more frequently said “I don’t know”. Generalized trust was not related to children’s overall trust rate in the game, *p* = .23, nor was it associated with different baseline levels of trust, *p* = .73.

## Regression results of the effects of testing format on children’s trust decisions

To address whether different testing formats (online versus in-person) and unequal sample sizes for each testing format might have affected participant performance, we added testing format as a covariate in the regression models reported in the main manuscript. We found that the testing format was not significantly associated with participant’s economic trust decisions, *p*s > .23. Please see the regression estimates in Table S2 below.

**Table S2**

*The effects of condition, age, and baseline trust on trust decisions.*

|  | Step 1 | Step 2 | Step 3 |
| --- | --- | --- | --- |
| Intercept | 0.478*** | 0.483*** | 0.378*** |
|  | (0.028) | (0.028) | (0.040) |
| Testing Format | -0.032 | -0.041 | -0.041 |
|  | (0.036) | (0.036) | (0.034) |
| Condition | -0.076*** | -0.076*** | -0.076*** |
|  | (0.021) | (0.020) | (0.020) |
| Age |  | 0.028* | 0.029* |
|  |  | (0.012) | (0.011) |
| Condition × Age |  | -0.021 | -0.021 |
|  |  | (0.012) | (0.012) |
| Baseline |  |  | 0.081*** |
|  |  |  | (0.023) |
| *R^2^* | 0.040 | 0.056 | 0.144 |

*Notes*. Standardized estimates (beta) reported with standard errors in paratheses. * *p* < .05, ** *p* < .01, *** *p* < .001

## Regression results of the associations between post-hoc identification and **children’s trust decisions**

We conducted additional analyses to assess whether being able to correctly report the partners’ trustworthiness after the task is related to children’s trust decisions during the game.

Results showed age differences in post-hoc identification: 36% 6-year-olds, 63% 7-year-olds, 44% 8-year-olds, 77% 9-year-olds, 77% 10-year-olds and 93% 11-year-olds correctly identified the trustworthy partner post-hoc. An exploratory logistic regression confirmed that older children were significantly more likely to correctly identify partner trustworthiness, *χ*²(1) = 8.26, *p* = .004, OR = 1.72, 95% CI [1.19, 2.48].

Building on our main regression model, we added post-hoc identification as a covariate and tested its interaction with age and testing format. Results suggest no significant main effects of correct reporting of trustworthiness after game, *p* = .14, interaction between correct reporting and age (centered), *p* = .59, or interaction between correct reporting and testing format, *p* = .99. These findings suggest no clear association between children’s behaviors in the repeated trust game with them correctly reporting the partners’ trustworthiness level after the game.

## Trust decisions over trial by age

To illustrate subtle age-related changes in behavior, here we show sharing decisions over trials by partner, broken down into 6 ages.

**Figure S1**

*Trust decisions over trial by age*

*Note.* Average proportion of children’s sharing towards the trustworthy and the untrustworthy partner over the course of 20 trials of repeated Trust Game, broken down by age.
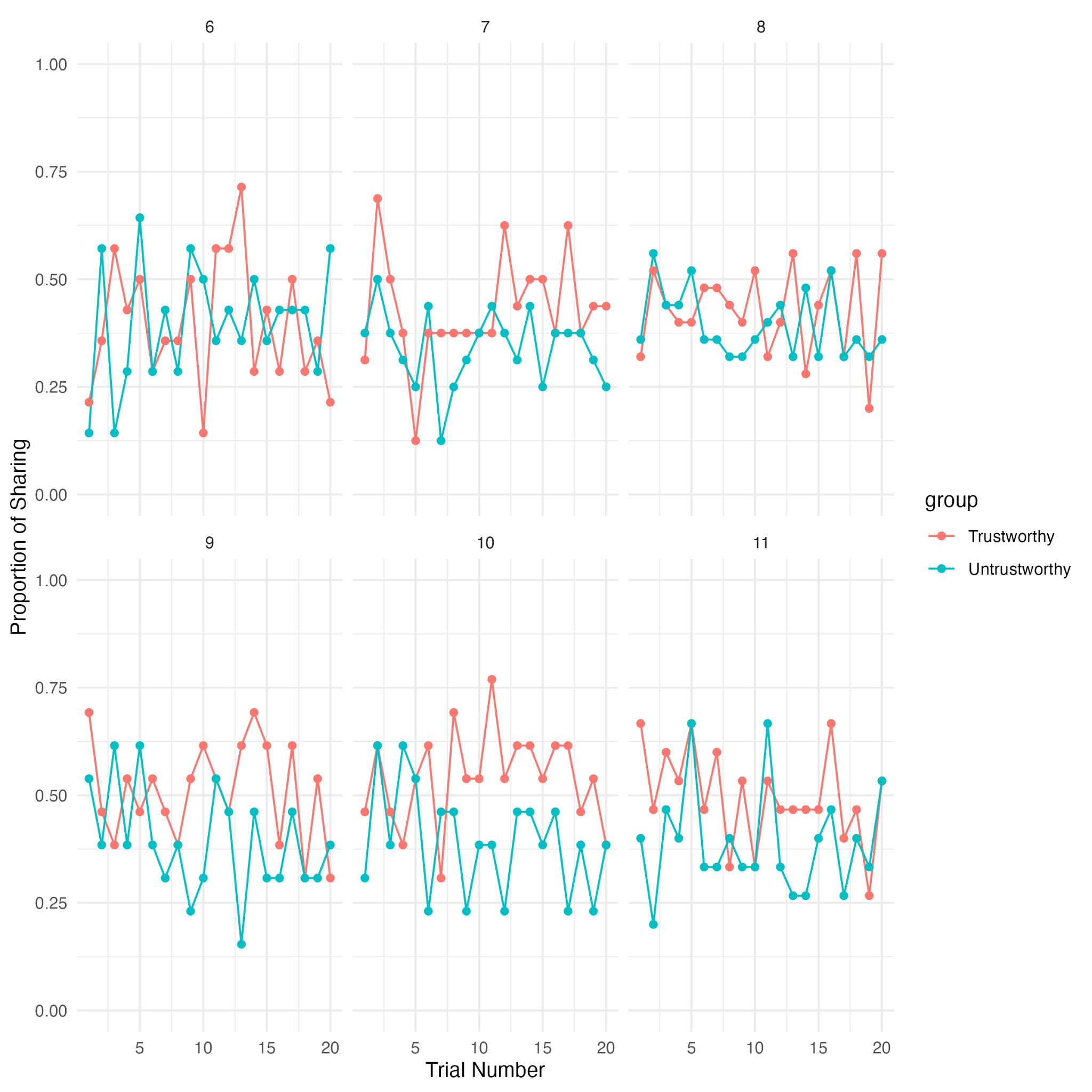


## Robustness check for alternative age grouping

In our main analyses, we created 3 age groups to estimate group-level parameters to investigate developmental differences in adapting to trustworthy and untrustworthy partners. To check the robustness of the age grouping, we replicated the reinforcement learning modeling with an alternative age grouping. Here, we split the age range into two, n = 55 children ages 6 to 8 years and n = 41 children ages 9 to 11 years, to see if the developmental pattern we reported in the main text is robust. We repeated the modeling analysis as described in the main manuscript.

Results suggest that the reinforcement learning model that included both individual trust expectations as priors and allowed for a decline in learning rate was still best fitting for both age groups. The 6-to 8-year-old group showed lower initial learning rate (*λ* = .06) with a moderate learning rate decline, while the 9-to 11-year-old group showed higher initial learning rate (*λ* = .24) with more rapid decline, estimates that corroborated the developmental trend we discussed in the main text.

## Robustness checks that exclude the first two trials (RL and GLMM)

In our experimental design, the first two trials with each partner were chosen to be congruent, a decision that increases control but might introduce anchoring effects to participants’ behaviors. To check the robustness of our modeling and to address the concern with anchoring, we run the following robustness checks that exclude data from the first two trials with each partner and report them below.

For the GLMM analyses of by-trial decisions, we used the same generalized linear mixed-effects model as reported in the main text that predicted trust decisions from partner trustworthiness, trial, age, and their interactions, with random intercepts for participants and random slopes for partner type. The only difference is that data from the first two trials with each partner is removed from this analysis. We found that the regression estimates are similar to the ones we found in the main analysis with full dataset, although the main effect of age attenuated, *p* = 14. This finding suggests that the two initial congruent trials did not drive the observed patterns of children’s economic trust behaviors.

For the reinforcement learning models, we replicated the main analysis with the data from all trials except for the first two trials. Again, the model with individual trust priors and allows for learning rate decline was best-fitting for all three age groups.

## Reinforcement learning model estimates

Here, we report reinforcement learning model estimates by age cohort and model type to provide more detailed information on modeling and model comparisons.

**Table S3**

*Reinforcement learning model estimates*

| Age | Model | *λ* | decayPar | *θ* | LL | BIC | BIC diff |
| --- | --- | --- | --- | --- | --- | --- | --- |
| 6-7 | 1 | 0.504  (0.152) | / | 0.206  (0.063) | 1657.556 | 1663.714 | 5.997 |
|  | 2 | 0.018  (0.006) | / | 0.963  (0.101) | 1610.287 | 1619.524 | 53.267 |
|  | 3 | 0.505  (0.152) | 5.88e-05  (6.38e-06) | 0.206  (0.063) | 1657.556 | 1666.794 | 5.997 |
|  | 4 | 0.068  (0.052) | 0.973  (0.862) | 0.982  (0.103) | 1609.634 | 1621.950 | 53.920 |
| 8-9 | 1 | 0.361  (0.170) | / | 0.257  (0.066) | 2097.442 | 2103.806 | 9.725 |
|  | 2 | 0.015  (0.005) | / | 1.071  (0.093) | 2027.623 | 2037.169 | 79.544 |
|  | 3 | 0.360  (0.170) | 9.57e-07  (6.39e-06) | 0.257  (0.066) | 2097.442 | 2107.736 | 8.977 |
|  | 4 | 0.122  (0.032) | 4.999  (6.39e-06) | 1.062  (0.088) | 2025.733 | 2038.460 | 81.434 |
| 10-11 | 1 | 0.428  (0.128) | / | 0.430  (0.075) | 1530.839 | 1536.937 | 21.811 |
|  | 2 | 0.052  (0.013) | / | 0.824  (0.103) | 1518.869 | 1528.017 | 33.781 |
|  | 3 | 0.428  (0.128) | 2.74e-06  (6.37e-06) | 0.430  (0.075) | 1530.839 | 1539.986 | 21.811 |
|  | 4 | 0.314  (0.051) | 4.999  (6.37e-06) | 0.792  (0.097) | 1515.427 | 1527.623 | 37.223 |

*Note*. Model 1: no prior, no decay; Model 2: prior, no decay; Model 3: no prior, decay; Model 4: prior & decay. Best parameter estimates and Hessian standard error in parentheses. BIC difference was calculated by subtracting model BIC from a completely random model BIC.

## Robustness check for modeling estimates using posterior predictive checks

Posterior predictive checks were conducted separately for each age cohort using the cohort-level best-fitting reinforcement learning model parameter estimates. For each cohort, we simulated 200 datasets and compared simulated and observed trial-by-trial trust rates, see Figure S2 below.

**Figure S2**

*Observed and simulated behavior by age cohort.*

*Note*. Observed behaviors are plotted in solid lines; 95% CI of behavior simulated from best fitting model parameters are plotted in the shaded area. Age cohort 1: 6- to 7-year-olds; age cohort 2: 8- to 9-year-olds); age cohort 3: 10- to 11-year-olds.
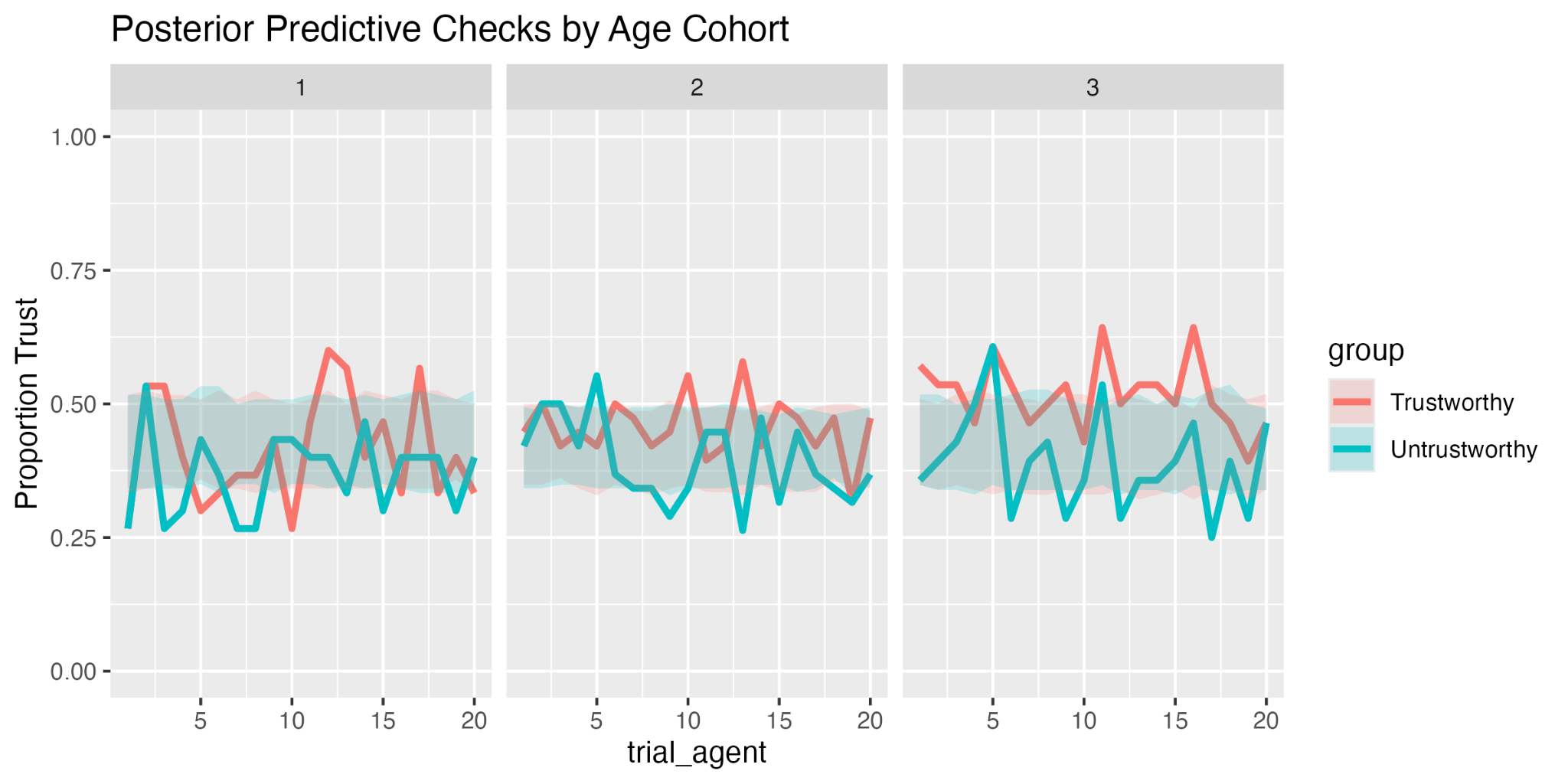

Supplement: Supplementary file 1 — Supporting File 1: desc70205‐sup‐0001‐SuppMat.docx [file DESC-29-e70205-s001.docx]
