# Supplementary material for: Trusting Wisely? Developmental Changes in How Children Learn and Adapt to Partner Trustworthiness
Source: Dev Sci. 2026 Apr 26;29:e70205. doi: 10.1111/desc.70205 (PMC13111782; doi:10.1111/desc.70205)
Supplement: Supplementary file 2 — Supporting File 2: desc70205‐sup‐0002‐tableS1‐S3.docx [file DESC-29-e70205-s002.docx]

## **Table S1.** *Participant demographic information*

| **Variable** | **Categories** | **Frequency** | **Percentage** |
| --- | --- | --- | --- |
| Gender |  |  |  |
|  | Girl | 47 | 48.96% |
|  | Boy | 48 | 50% |
|  | Did not report | 1 | 1.04% |
| Race |  |  |  |
|  | Asian or Asian American | 10 | 10.41% |
|  | Black or African American | 9 | 9.38% |
|  | Multiracial | 11 | 11.46% |
|  | Native American or Pacific Islander | 1 | 1.04% |
|  | White or European American | 62 | 64.58% |
|  | Other | 3 | 3.13% |
| Ethnicity |  |  |  |
|  | Hispanic or Latino | 14 | 14.58% |
|  | Not Hispanic or Latino | 82 | 85.42% |
| Household Income |  |  |  |
|  | Up to $39,999 | 6 | 6.25% |
|  | $40,000 to $69,999 | 18 | 18.75% |
|  | $70,000 to $99,999 | 12 | 12.5% |
|  | $100,000 to $149,999 | 20 | 20.83% |
|  | $150,000 to $ 199,999 | 15 | 15.63% |
|  | $200,000 or more | 19 | 19.79% |
|  | Prefer not to say | 6 | 6.25% |
| Caregiver Education |  |  |  |
|  | Some high school or less | 0 | 0% |
|  | Highschool | 4 | 4.17% |
|  | Some College (1-3 years, Associate’s degree) | 9 | 9.38% |
|  | 4 year degree (Bachelor’s degree) | 25 | 26.04% |
|  | Graduate degree (Master’s, doctoral, or professional degree) | 58 | 60.42% |
| Testing location |  |  |  |
|  | Local parks | 9 | 9.38% |
|  | Local museums | 34 | 35.43% |
|  | Online | 53 | 55.21% |

**Table S2**

*The effects of condition, age, and baseline trust on trust decisions.*

|  | Step 1 | Step 2 | Step 3 |
| --- | --- | --- | --- |
| Intercept | 0.478*** | 0.483*** | 0.378*** |
|  | (0.028) | (0.028) | (0.040) |
| Testing Format | -0.032 | -0.041 | -0.041 |
|  | (0.036) | (0.036) | (0.034) |
| Condition | -0.076*** | -0.076*** | -0.076*** |
|  | (0.021) | (0.020) | (0.020) |
| Age |  | 0.028* | 0.029* |
|  |  | (0.012) | (0.011) |
| Condition × Age |  | -0.021 | -0.021 |
|  |  | (0.012) | (0.012) |
| Baseline |  |  | 0.081*** |
|  |  |  | (0.023) |
| *R^2^* | 0.040 | 0.056 | 0.144 |

*Notes*. Standardized estimates (beta) reported with standard errors in paratheses. * *p* < .05, ** *p* < .01, *** *p* < .001

**Table S3**

*Reinforcement learning model estimates*

| Age | Model | *λ* | decayPar | *θ* | LL | BIC | BIC diff |
| --- | --- | --- | --- | --- | --- | --- | --- |
| 6-7 | 1 | 0.504  (0.152) | / | 0.206  (0.063) | 1657.556 | 1663.714 | 5.997 |
|  | 2 | 0.018  (0.006) | / | 0.963  (0.101) | 1610.287 | 1619.524 | 53.267 |
|  | 3 | 0.505  (0.152) | 5.88e-05  (6.38e-06) | 0.206  (0.063) | 1657.556 | 1666.794 | 5.997 |
|  | 4 | 0.068  (0.052) | 0.973  (0.862) | 0.982  (0.103) | 1609.634 | 1621.950 | 53.920 |
| 8-9 | 1 | 0.361  (0.170) | / | 0.257  (0.066) | 2097.442 | 2103.806 | 9.725 |
|  | 2 | 0.015  (0.005) | / | 1.071  (0.093) | 2027.623 | 2037.169 | 79.544 |
|  | 3 | 0.360  (0.170) | 9.57e-07  (6.39e-06) | 0.257  (0.066) | 2097.442 | 2107.736 | 8.977 |
|  | 4 | 0.122  (0.032) | 4.999  (6.39e-06) | 1.062  (0.088) | 2025.733 | 2038.460 | 81.434 |
| 10-11 | 1 | 0.428  (0.128) | / | 0.430  (0.075) | 1530.839 | 1536.937 | 21.811 |
|  | 2 | 0.052  (0.013) | / | 0.824  (0.103) | 1518.869 | 1528.017 | 33.781 |
|  | 3 | 0.428  (0.128) | 2.74e-06  (6.37e-06) | 0.430  (0.075) | 1530.839 | 1539.986 | 21.811 |
|  | 4 | 0.314  (0.051) | 4.999  (6.37e-06) | 0.792  (0.097) | 1515.427 | 1527.623 | 37.223 |

*Note*. Model 1: no prior, no decay; Model 2: prior, no decay; Model 3: no prior, decay; Model 4: prior & decay. Best parameter estimates and Hessian standard error in parentheses. BIC difference was calculated by subtracting model BIC from a completely random model BIC.
